# Genome-Wide Association Studies Reveal Novel Loci for Herbivore Resistance in Wild Soybean (*Glycine soja*)

**DOI:** 10.3390/ijms23148016

**Published:** 2022-07-20

**Authors:** Haiping Du, Rui Qin, Haiyang Li, Qing Du, Xiao Li, Hui Yang, Fanjiang Kong, Baohui Liu, Deyue Yu, Hui Wang

**Affiliations:** 1 Innovative Center of Molecular Genetics and Evolution, School of Life Sciences, Guangzhou University, Guangzhou 510405, China; yanghui_90@gzhu.edu.cn (H.Y.); kongfj@gzhu.edu.cn (F.K.); liubh@gzhu.edu.cn (B.L.); 2 National Center for Soybean Improvement, National Key Laboratory of Crop Genetics and Germplasm Enhancement, Nanjing Agricultural University, Nanjing 210095, China; rui.qin@geneseeq.com (R.Q.); haiyangli2021@163.com (H.L.); duqing@vazyme.com (Q.D.); xiao_li@njau.edu.cn (X.L.); dyyu@njau.edu.cn (D.Y.)

**Keywords:** wild soybean (*Glycine soja* Sieb. & Zucc.), resistance to insects, common cutworm, reactive oxygen species, *GsRbohA1*

## Abstract

The production of soybean [*Glycine max* (L.) Merr.] is seriously threatened by various leaf-feeding insects, and wild soybean [*Glycine soja* Sieb. & Zucc.] has a greater resistance capacity and genetic diversity. In this study, a natural population consisting of 121 wild soybean accessions was used for detecting insect resistance genes. The larval weight (LW) of the common cutworm (CCW), the resistance level (RL) and the index of damaged leaf (IDL) were evaluated as resistance indicators to herbivores. An association synonymous SNP AX-94083016 located in the coding region of the respiratory burst oxidase gene *GsRbohA1* was identified by genome-wide association study (GWAS) analyses. The overexpression of *GsRbohA1* in soybean hairy roots enhanced resistance to CCW. One SNP in the promoter region cosegregated with AX-94083016 contributing to soybean resistance to CCW by altering *GsRbohA1* gene expression and reactive oxygen species (ROS) accumulation. Two major haplotypes, *GsRbohA1^A^* and *GsRbohA1^G^*, were identified based on the SNP. The resistant haplotype *GsRbohA1^A^* predominates in wild soybeans, although it has been gradually lost in landraces and cultivars. The nucleotide diversity around *GsRbohA1* is much lower in landraces and cultivars than in its ancestors. In conclusion, a new resistant haplotype, *GsRbohA1^A^,* was identified in wild soybean, which will be a valuable gene resource for soybean insect resistance breeding through introducing into improvement lines, and it offers a strategy for exploring resistance gene resources from its wild relatives.

## 1. Introduction

Soybean [*Glycine max* (L.) Merr.] provides abundant protein for the human diet and is one of the most important oil crops. However, the production of soybean is seriously threatened by various leaf-feeding insects [1,2,3]. Among them, common cutworm [*Spodoptera litura* Fabricius] (CCW) is the main harmful insect in southern soybean regions in China. Although some resistant accessions, quantitative trait loci (QTLs) and genes have recently been identified in soybean, herbivore damage is still a particular problem for field production due to genetic bottlenecks [4,5,6,7,8,9].

Annual wild soybean [*Glycine soja* Sieb. & Zucc.], the wild relative of cultivated soybean, has a large degree of genetic diversity and serves as a resource for breeders to discover elite defensive genes. Oki et al. detected two QTLs, *qRslx3* and *qRslx4,* related to CCW resistance in wild soybean [10]. Moreover, some resistance alleles to soybean cyst nematodes and aphids have been identified in wild accessions [11,12]. Therefore, wild soybean accessions are good choices for detecting resistance resources.

Antibiosis and antixenosis are two types of plant resistance to insects. The former comprises a series of responses when attacked to prevent insect fitness and is usually measured by insect development, such as the larval weight, pupal weight and larval stage duration [3,13,14,15,16]. Antixenosis is usually constitutive, acting as a physical barrier to interfere with herbivore feeding, attachment and oviposition and is measured by certain traits of the host plants, such as the pubescence density, pubescence length, defoliation and damage leaf percentage [6,14,17,18,19].

Genome-wide association studies (GWAS) are considered an efficient strategy for discovering loci and genes in plants and have recently been successfully applied in various species [20,21,22]. GWAS of resistance to CCW, beet armyworm, Mexican bean beetle, potato leafhopper, soybean aphid, soybean looper and other insects have been reported in cultivated soybeans [3,23,24]. In addition, a GWAS of resistance to soybean cyst nematodes has been conducted in wild soybean [25]. However, wild soybean accessions have not been used to dissect resistance genes to CCW by GWAS.

Reactive oxygen species (ROS) play an important role in plant defense responses [26]. NADPH oxidase genes of plants, abbreviated *Rboh* (respiratory burst oxidase homolog), are closely related to ROS metabolism under stress conditions. The rice gene *OsRbohA* was the first *Rboh* gene identified in plants [27], after which, multiple *Rboh* genes were identified, including in *Arabidopsis*, tobacco; *Medicago truncatula*, tomato; and other crops [28,29,30,31].

Moreover, Zeng et al. identified 17 *Rboh* genes in soybean that show a close relationship with ten *AtRboh* members [32]. Four *Rbohs* were reported to be involved in pathogenic development in soybean [33]. *Rboh*-dependent ROS production has also been studied in various plant species. In *Nicotiana attenuate*, silencing *NaRbohD* significantly reduces ROS levels and the transcript levels of defense genes after oral secretion (OS) elicitation [34]. Among the ten *AtRboh* genes, *AtRbohD* and *AtRbohF* are required for ROS accumulation in the plant defense response [35]. In particular, *AtRbohF* interacts closely with intracellular oxidative stress to fine-tune dynamic metabolic responses during infection [36].

In this study, three traits associated with resistance to CCW in a wild soybean population of 121 accessions was evaluated. Genome-wide association analyses were performed with a 355 K SoySNP array and three resistance traits to herbivores. We then characterized the function of the candidate gene *GsRbohA1* in regulating soybean resistance to CCW through soybean hairy root transformation. The functional difference between haplotypes *GsRbohA1^A^* and *GsRbohA1^G^* was verified by measuring the gene expression and ROS accumulation in representative lines. To understand the domestication pattern of the resistance haplotype *GsRbohA1^A^*, we analyzed the genetic diversity and *F*_st_ in wild, landrace and cultivar populations. The objective of this study was to identify resistance gene resources in wild soybeans to improve cultivar resistance to insects.

## 2. Results

### 2.1. Phenotypic Variation in the Population

**Figure 1:**
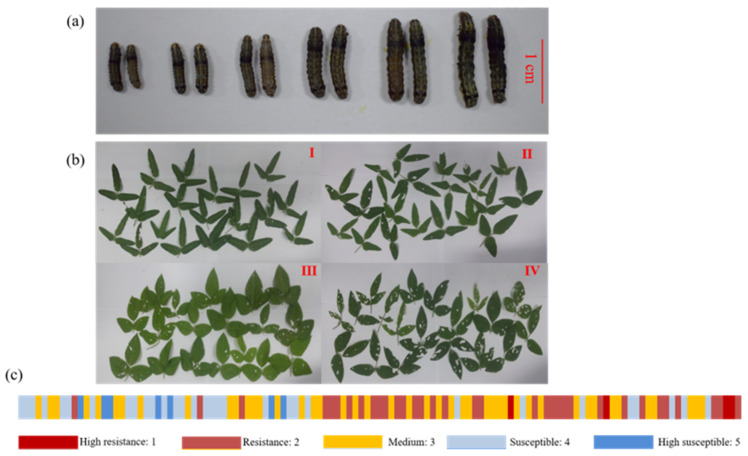
Four resistance factors to common cutworm (CCW) used in this study. (**a**) CCW larvae were fed different wild soybeans for 7 days. The larval weight was measured, and the red scale bar represents 1 cm. (**b**) Index of damaged leaf according to the loss of leave damaged by insects at levels I, II, III and IV. (**c**) The resistance level distribution of the wild soybean population according to the larval weight.

In this study, the larval weight (LW) and resistance level (RL) were used to evaluate wild soybean resistance to CCW as antibiosis factors, and the index of damaged leaf (IDL) was used to assess resistance to herbivores as an antixenosis factor (Figure 1). These resistance indicators were investigated for the years 2014 and 2016. Plants grown in 2016 were more resistant to insects regardless of being in the laboratory (LW) or filed (IDL) at the population level (Supplementary Table S2 and Figure S1a,c). However, the resistance level of individual accession maintained good consistency within two years in the population (Figure S1b,d). The Pearson correlation coefficients between two years were 0.41 (*p* = 4.49 × 10^−6^, two side), 0.46 (*p* = 2.71 × 10^−7^, two side) and 0.48 (*p* = 6.11 × 10^−8^, two side) for LW, RL and IDL, respectively.

**Figure 2:**
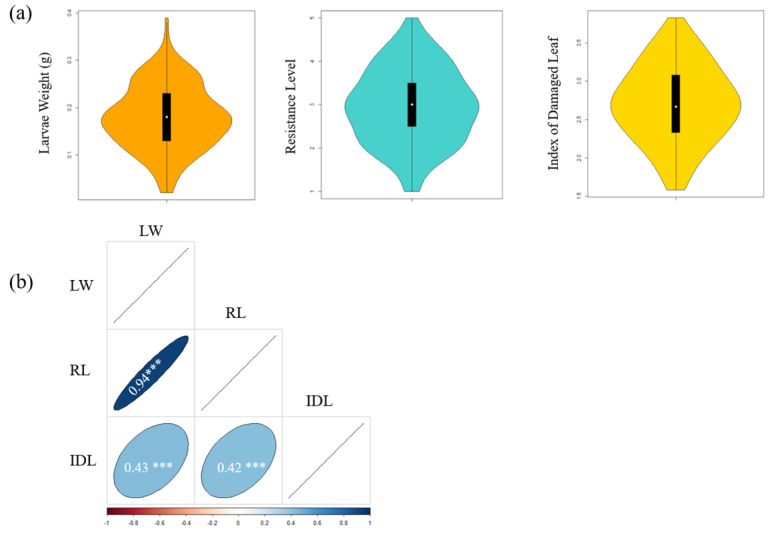
Phenotype distribution and correlation. (**a**) Violin plots of three traits. (**b**) Pearson correlation between the larval weight (LW), resistance level (RL) and index of damaged leaf (IDL). The number in the color circle represents the correlation coefficient; *** *p* < 0.001.

All three phenotypic values presented a continuous distribution (Figure 2a and Supplementary Figure S2), with variation ranging from 0.0050–0.5433 g, 1.67–5.00 and 1.33–4.00 for LW, RL and IDL, respectively (Supplementary Table S3). The ANOVA results indicated that all three traits were significantly affected by the genotype and environment (Supplementary Table S3).

The correlation coefficients between herbivore resistance traits were calculated based on the mean values of the two years (Figure 2b). The results indicated that soybean antibiosis was associated with antixenosis and that they might, to some extent, be controlled by the same locus.

### 2.2. Quality Control of SNP Markers and Population Structure

A total of 292,053 high-quality SNP markers from 105 wild soybeans were used for genetic analyses; 110,117 of the 292,053 SNPs (37.70%) with MAF ≥ 0.05 were used for further GWAS analyses.

**Figure 3:**
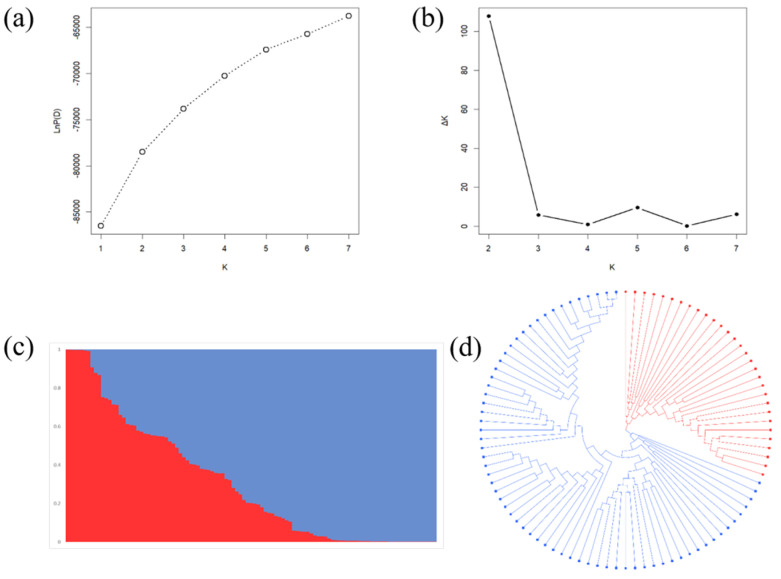
The population structure of 105 wild soybean accessions. (**a**,**b**) LnP (D) and Δk based on 20 runs of STRUCTURE analysis. (**c**) The population structure at K = 2 estimated by STRUCTURE. There are two colored segments, with each representing the percentage belonging to each subpopulation. (**d**) A neighbor-joining tree of the 105 accessions divided into two subpopulations.

Population structure has a great influence on the results of association analyses. STRUCTURE and NJ tree analyses were conducted to evaluate the population structure and relatedness among 105 wild soybeans. The STRUCTURE analysis showed that LnP (D) increased with increasing K and had the highest slope from K = 1 to K = 2 (Figure 3a). The ad hoc quantity (ΔK) showed a clear peak at K = 2, indicating that the population can be clustered into two subpopulations (Figure 3b,c).

However, the peak value of ΔK at K = 2 is around 110, implying that the population structure is weak (Figure 3b). This may be due to the fact that the population analyzed here is only made up of wild soybean varieties, which have a close genetic relationship with each other. Then, the results were further supported by NJ tree analysis (Figure 3d). Therefore, the Q-matrix at K = 2 calculated by STRUCTURE was used for subsequent GWAS analyses.

### 2.3. GsRbohA1 Is a Candidate Gene for Wild Soybean Resistance to CCW

**Figure 4:**
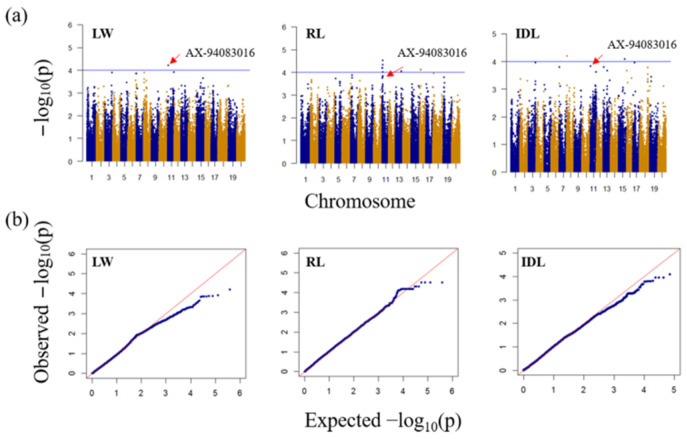
Genome-wide association analysis (GWAS) of the larval weight (LW), resistance level (RL) and index of damaged leaf (IDL) with the mean values of two years. (**a**) Manhattan plots describe the GWAS results using 110,117 SNPs. Horizontal lines represent an interesting threshold (−log_10_*p* = 4.00), and AX-94083016 is indicated. (**b**) Quantile–quantile (qq) plots for three traits.

The mixed linear model (MLM) with Q + K used for GWAS in this study was somewhat strict for the three resistance traits (Figure 4b); therefore, −log_10_*p* = 4.00 was used as a suggestive threshold (Figure 4a). There were 1, 34 and 2 SNPs significantly associated with the LW, RL and IDL, respectively (Supplementary Table S4). Among them, SNP AX-94083016 on chromosome 11 (physical position is 1448659 *Glycine max* Wm82.a1.v1.1) was the only association marker with LW.

Then, we found that SNP AX-94083016 was also associated with RL and IDL with −log_10_*p* values of 3.79 and 3.82, respectively (Figure 4a). Additionally, a significant SNP cluster containing seven SNPs (physical position from 1373761 to 1505923 *Glycine max* Wm82.a1.v1.1) associated with RL in the LD region of AX-94083016 on chromosome 11 was detected (Figure 4a). Therefore, we selected AX-94083016 as candidate marker for further analysis first.

AX-94083016 is a synonymous SNP located in the coding regions of *Glysoja_004816*, which encodes the respiratory burst oxidase protein, also known as NADPH oxidase protein, which is involved in the plant defense response to biotic stresses by mediating ROS production. Therefore, *Glysoja_004816* is a candidate gene regulating soybean resistance to CCW. *Glysoja_004816* is homologous to the cultivar *RbohA* gene *Glyma.11G020700*; it is named *GsRbohA1*.

### 2.4. GsRbohA1 Positively Increases Soybean Resistance to CCW

**Figure 5:**
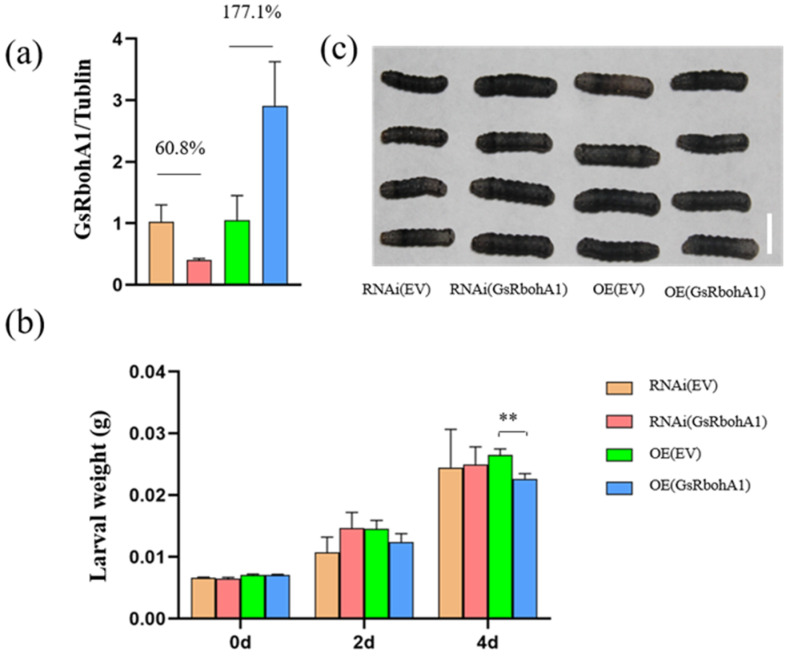
Functional characterization of *GsRbohA1*. (**a**) The transcriptional level of *RbohA1* in hairy roots overexpressing *GsRbohA1* (OE (*GsRbohA1*)), RNA interference for *GsRbohA1* (RNAi (*GsRbohA1*)), the overexpression empty vector (OE (EV)) and the RNA interference empty vector (RNAi (EV)). The error bars represent standard deviations, *n* = 3. (**b**) larval weight of common cutworm (CCW) before feeding and at 2 days and 4 days after feeding on soybean hairy roots. Error bars represent the standard deviations, *n* ≥ 12, and statistical significance was detected by a two-tailed *t* test. ** *p* < 0.01. (**c**) CCW larvae feeding on soybean hairy roots for 4 days; the scale bar represents 1 cm.

To investigate whether *GsRbohA1* regulates soybean resistance to CCW, we cloned the coding sequence of *GsRbohA1* from one resistant line and one susceptible line. As no amino acid changes were detected in the coding regions between the two lines, the coding sequence from the susceptible line was used. The overexpression plasmid pMDC83-*GsRbohA1*, RNAi plasmid pB7-*GsRbohA1* and the corresponding empty vectors pMDC83-EV and pB7-EV were transformed into soybean hairy roots. The *RbohA1* expression decreased by 60.8% but increased by 177.1% in hairy roots expressing the RNAi and overexpression plasmids compared with the corresponding control groups, respectively (Figure 5a).

However, ROS staining analyses of the hairy roots showed no significant difference between the RNAi and control samples; staining was significantly deeper in the overexpression hairy roots than in the control roots (Figure S3). Hairy roots were used to feed CCW larvae; after 4 days, the larval weight after feeding on the overexpression hairy roots was significantly lower than that of the control group, whereas there was no significant difference between the RNAi and control groups (Figure 5b,c). These results show that *GsRbohA1* regulates soybean resistance to CCW by mediating ROS accumulation.

### 2.5. An SNP in the Promoter Alters GsRbohA1 Expression and ROS Accumulation

**Figure 6:**
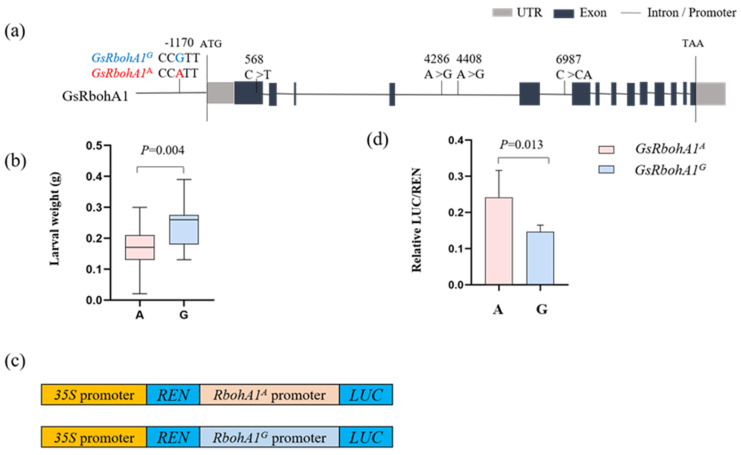
Resistance haplotype GsRbohA1A has stronger transcriptional activity than GsRbohA1G. (**a**) Structural variations of GsRbohA1 in the wild soybean population used in this study. The position number starts counting at ATG, including the intron region. (**b**) The larval weight distribution of GsRbohA1A and GsRbohA1G haplotypes. (**c**) Constructs used for the transient transfection assay. (**d**) Luciferase activity under the control of the promoters GsRbohA1A and GsRbohA1G, respectively. The error bars represent the standard deviations, n = 4; statistical significance was detected by a two-tailed t-test. Two independent assays showed similar results.

To investigate the functional allelic variation of *GsRbohA1*, we further analyzed the *GsRbohA1* SNP using high-density SNPs described previously in the wild soybean population [37]. Five new SNPs associated with herbivore resistance were identified. One is in a Myb-binding core motif (AACGG) in the promoter region, three are located in the intron region, and one is present in the first exon region, which does not change the amino acid sequence (Figure 6a). The SNP in the promoter cosegregated with SNP AX-94083016 in more than 95% of lines in the wild soybean population and may affect the expression of *GsRbohA1,* resulting in resistance differences. Therefore, we further characterized the function of the two haplotypes *GsRbohA1^A^* and *GsRbohA1^G^* based on the promoter SNP (Figure 6a).

CCW larvae feeding on *GsRbohA1^A^* lines were significantly lighter in weight than larvae feeding on *GsRbohA1^G^* lines among the wild soybean population (*t* test, *p* = 0.004) (Figure 6b). Hence, we refer to *GsRbohA1^A^* as the resistance haplotype. To explore whether *GsRbohA1^A^* and *GsRbohA1^G^* have different transcriptional activities, a dual-luciferase transient expression assay using the *GsRbohA1^A^* or *GsRbohA1^G^* promoter fused to the LUC reporter was performed (Figure 6c).

The relative LUC/REN value showed that promoter *GsRbohA1^A^* has stronger transcriptional activity than *GsRbohA1^G^* (*t* test, *p* = 0.013) (Figure 6d). Then, four lines for each haplotype were randomly selected for investigating the *GsRbohA1* expression and ROS accumulation. The results showed that the *GsRbohA1* expression was over twofold and threefold higher in *GsRbohA1^A^* than in *GsRbohA1^G^* under the control and CCW induction conditions, respectively (Figure S4a).

In addition, ROS accumulation in *GsRbohA1^A^* was higher than that in *GsRbohA1^G^* under both control and CCW induction conditions (Figure S4b). These results indicate that haplotype *GsRbohA1^A^* possesses stronger transcriptional activity and confers strong resistance to CCW by increasing the *GsRbohA1* expression level and ROS accumulation in wild soybean.

### 2.6. The Resistance Allele of GsRbohA1 Was Gradually Lost in Soybean Cultivars during Domestication and Improvement

**Figure 7:**
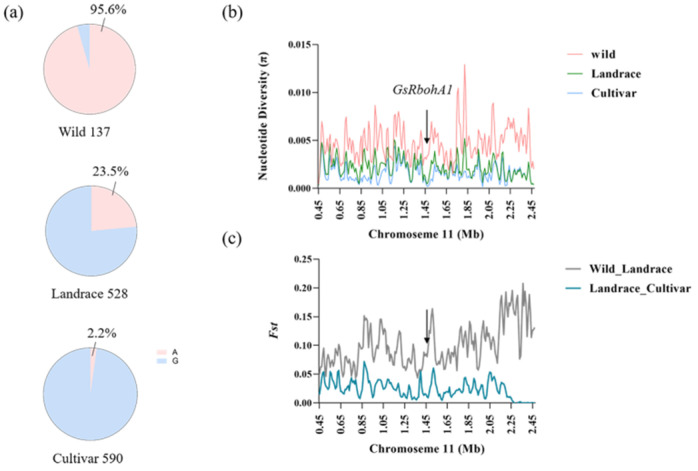
The resistance haplotype of *GsRbohA1* was gradually lost during soybean domestication. (**a**) Proportion of *GsRbohA1^A^* and *GsRbohA1^G^* in wild, landrace and cultivated soybeans. (**b**) Nucleotide diversity (π) analysis of the 2 Mb region flanking *GsRbohA1*. (**c**) Level of genetic differentiation (*F*_st_) across 2 Mb regions flanking *GsRbohA1* between subspecies.

Interestingly, we found that, for the wild soybean population we used, most varieties were the *GsRbohA1^A^* haplotype (108/121), and we examined the haplotypes in 1295 resequenced accessions, including the 121 wild lines we used. The proportion of *GsRbohA1^A^* was 95.6% in the wild population, decreased to 23.5% in landraces and was only 2.2% in cultivars (Figure 7a). These results indicate that the resistance allele *GsRbohA1^A^* was gradually lost from wild to landrace and cultivar during domestication and improvement.

We further compared the nucleotide diversity (π) and the level of genetic differentiation (*F*_st_) across chromosome 11 in the 2-Mb genomic region spanning *GsRbohA1* between different subspecies in the 1295 diversity panel described above and observed severe loss of nucleotide diversity around *GsRbohA1* in landraces and cultivars (Figure 7b,c). The *F*_st_ level between wild and landrace plants was medium, also indicating that the loss of nucleotide diversity around *GsRbohA1* was a gradual process (Figure 7c).

## 3. Discussion

### 3.1. Correlation between Traits

All three traits used in this study showed continuous variation (Figure S2), thereby, indicating that they are all quantitative traits controlled by multiple genes. Among them, LW was greatly affected by both the host plants and individual larvae differences (Figure S1a), and few QTLs related to insect resistance were stably identified in previous studies [14,15,16]. To reduce the environmental influence on LW, we converted it to five levels of RL, which is more powerful for identifying association signals (Figure 4a).

Unlike LW, which is an antibiosis index measured by CCW force feeding in the laboratory, IDL is an antixenosis index measured based on the damaged area of leaves caused by multiple species of insects under field conditions. A positive correlation between the two traits indicated a correlation between antibiosis and antixenosis of host plants (Figure 2b). Previously, antibiosis (*CCW-1*) and antixenosis (*qRslx1*) QTLs for CCW were identified within a close location on chromosome 7 [5,6]. In this study, we also identified SNP AX-94083016, which was associated with both LW and IDL (Figure 4a).

### 3.2. Functional Redundancy of GsRbohA

Approximately 75% of genes are present with multiple copies in the soybean genome, and functional redundancy is a common phenomenon [38]. Regarding *RbohA*, four copies were identified in the wild soybean genome. In this study, a decrease in *GsRbohA1* expression did not significantly affect the ROS accumulation or resistance to herbivores in the RNAi hairy roots compared with the control groups (Figure 5b,c and Figure S3), which may be due to the functional redundancy of genes. Thus, double or even multiple mutants of *GsRbohA* genes are needed for further characterization of the gene.

### 3.3. GsRbohA1^A^ Is an Elite Allele for Improving Cultivated Soybean Resistance to Herbivores

In this study, we identified and characterized a wild soybean *Rboh* gene that positively regulates resistance to CCW by increasing the gene expression and ROS accumulation (Figure 5 and Figure S3). This is consistent with the fact that the concentration of intracellular ROS is critical for plant growth and development: it is low under normal conditions to promote cell growth but increases dramatically in the presence of a variety of environmental challenges to initiate the defense response [39,40,41,42].

We identified a resistance promoter haplotype, *GsRbohA1^A^*, that promotes gene expression and ROS accumulation, thereby, contributing to resistance to CCW in wild soybean (Figure 6 and Figure S4). Although *GsRbohA1^A^* is predominant in the wild population, it is rare in cultivated soybean (Figure 7a). As reported, during domestication and improvement processes, approximately half of the genetic diversity was lost in landraces and cultivars. In particular, half of the annotated resistance-related sequences in wild soybean were lost in both landraces and cultivars [43,44,45]. Therefore, introducing the resistance allele *GsRbohA1^A^* of wild ancestors into cultivated lines is a feasible strategy to improve cultivar resistance.

## 4. Methods and Materials

### 4.1. Plant Materials and Field Growth

All 121 wild accessions used in this study were provided by the National Center for Soybean Improvement (Nanjing, China) [46]. We evaluated the CCW resistance of populations over 2 years (2014 and 2016) at Jiangpu Experimental Station of Nanjing Agricultural University (Nanjing, China). The materials were grown in hill plots in a randomized complete block design with three replications; each replication contained four hills with 20 plants for each accession. The hills were planted every 50 cm along rows spaced 50 cm apart for the same accession and 100 cm along rows spaced 100 cm apart between accessions. A bamboo pole was placed close to each hill to support twinning stems. The field was surrounded by a nylon mesh to exclude hares and other animals, and no chemical insecticides were used during the soybean growth period.

### 4.2. Larval Weight

Force-feeding experiments were conducted as previously described by Fan et al. [47]. Wild soybeans were grown for approximately 45 d after germination, and the upper, fully expanded leaves were selected for feeding CCW larvae in the laboratory. Five 2-instar CCW larvae were raised in a culture tank with fresh leaves for 7 d; fresh leaves were replaced every 2 d during this process. The larvae were weighed on the seventh day after feeding. Bioassays were replicated in the same manner as the field experiments.

To evaluate the function of *GsRbohA1*, force-feeding experiments were conducted as described above. Five 2-instar CCW larvae were raised in a culture tank with fresh soybean hairy roots for 4 days; the fresh hairy roots were replaced at 2 d after feeding. The larvae were weighed before feeding and 2 days and 4 days after feeding. Each experiment was repeated four times. Microsoft Excel 2010 was used to statistically analyze the data.

### 4.3. Resistance Level

The materials were divided into five resistance levels based on the larval weight (LW), as previously described by Cui et al. [48]. LW ≤ M − 1.5S is the high resistance level (1); LW > M − 1.5S and ≤M − 0.5S is the resistance level (2); LW > M − 0.5S and ≤ M + 0.5S is the middle level (3); LW > M + 0.5S and ≤ M + 1.5S is the susceptible level (4); and LW ≥ M + 1.5S is the highly susceptible level (5). M represents the average, and S represents the standard deviation.

### 4.4. Index of Damaged Leaf

Plants are attacked by insect pests when grown naturally in the field, resulting in different percent defoliation ratings. The material index of damaged leaves was divided into four levels according to the number of infested leaves on a plant and the percentage of defoliation ratings, as follows: level 1: less than 30% of leaves were infected; level 2: 30% to 50% of leaves were infected, with an average percentage of defoliation ratings of less than 30%; level 3: 30% to 50% of leaves were infected, with more than 30% average defoliation ratings or 50% to 80% of the leaves were infected; and level 4: more than 80% of leaves were infected (Figure 1c).

### 4.5. Genome-Wide Association Analyses

A total of 292,053 single-nucleotide polymorphism (SNP) markers were genotyped in 105 of 122 wild soybean accessions as described previously (NJAU 355K SoyaSNP Array, https://www.soybase.org/projects/SoyBase.C2021.03.php, accessed on 19 April 2021) [46]. For the wild soybeans used in our study, Wang et al. previously calculated that the distance over which LD decays to half of its maximum value is 80 kb [46]. In this study, both genotype and phenotype data were available for only 104 accessions.

A total of 110,117 SNPs with minor allele frequencies (MAF) ≥ 0.05 were employed for further analyses. The population structure (Q) was measured with STRUCTURE 2.3.4, and the kinship matrix (K) was measured with TASSEL 5.2.2 software [49]. Genome-wide association analyses were performed based on a mixed linear model (MLM) with the Q + K model with TASSEL 5.2.2 software [49]. The Bonferroni threshold *p* ≤ (1/110117) or −log_10_*p* ≥ 5.04 was used to define significant association markers. SNPs of interest significantly (*p <* 0.0001 or −log_10_*p >* 4.00) associated with CCW resistance traits were defined as suggestive SNPs.

STRUCTURE 2.3.4 based on a Bayesian model was adopted to analyze the population structure and relatedness in wild soybeans. A length of the burn-in and MCMC (Markov chain Monte Carlo) of 100,000 each was set. The hypothetical number of subpopulations (K) was set from 1 to 7. For each data set, 20 runs were performed to quantify the amount of variation of the likelihood for each K. To detect the true number of clusters (K) in wild soybeans, the LnP (D) value (log likelihood of the data) and Δk value (an ad hoc statistic based on the rate of change in the log likelihood of the data) were used as reported by Evanno et al. [50]. TASSEL 5.2.2 was employed to construct a neighbor-joining (NJ) phylogenetic tree with 110,117 SNPs (MAF ≥ 0.05) with the default parameters.

### 4.6. Soybean Hairy Root Transformation

The *GsRbohA1* coding sequence was inserted into vector pMDC83 fused to GFP by homologous recombination under control of the CaMV 35S promoter to produce the overexpression plasmid pMDC83-*GsRbohA1*-GFP. A specific 420-bp fragment of *GsRbohA1* was amplified, and the fragment was inserted fused to GFP into vector pB7GWIWG2(II) by Gateway technology with a Clonase II kit (Invitrogen, Carlsbad, CA, USA) to produce RNAi plasmid pB7-*GsRbohA1*-GFP (Figure S5a). Both the full-length sequence and specific fragment were cloned from the cDNA of wild soybean accession zyd4179 (W11).

The empty vectors EV-pMDC83-GFP and EV-pB7-GFP were used as controls for the overexpression and RNAi plasmid, respectively. Hairy root transformation was performed using soybean cultivar accession Jack, which is known for having high transformation efficiency. Positive hairy roots were monitored for green fluorescence production under a microscope with a green fluorescence channel (Figure S5c). Four weeks after transformation, positive hairy roots were selected for the CCW feeding assay (Figure S5b). The gene expression was monitored in the fluorescent hairy roots by qRT–PCR (primers shown in Table S1).

### 4.7. Transient Expression Assay

To generate the *proRbohA1^A^-LUC* and *proRbohA1^G^-LUC* constructs, the 2-kb promoter sequences of *RbohA1* were amplified from zyd4976 (W99) and zyd4179 (W11), respectively. Then, the fragments were introduced into the pGreenII 0800-LUC vector. The *proRbohA1^A^-LUC* and *proRbohA1^G^-LUC* constructs were introduced into *Agrobacterium tumefaciens* strain GV3101 and transformed into tobacco leaf (*Nicotiana tabacum* cv. SamSun) via syringe infection. Three days after infection, the leaves were harvested for detecting REN and LUC luciferase activities. The values of LUC/REN represent the corresponding activities of promoters. We used four biological replicates.

### 4.8. ROS Staining

Zyd3314 (W117), zyd4976 (W99), zyd4698 (W121) and zyd4341 (W119) were chosen as representative *GsRbohA1^A^* lines, and zyd4353 (W95), zyd4179 (W11), zyd4157 (W103) and zyd4673 (W107) were chosen as representative *GsRbohA1^G^* lines for *GsRbohA1* expression and ROS accumulation analysis. CCW-induced (1 h after induction) and corresponding control samples were harvested. Three biological replicates were used for each treatment.

NBT staining was used to detect the level of ROS accumulation. First, NBT powder was dissolved in 10 mM potassium phosphate buffer (pH 7.8) to 0.5 mg/mL, and plant tissues were placed in the staining solution overnight at room temperature. Then, the stained tissues were placed in the eluent and boiled for 15 min at 90–95 °C. Finally, the hairy roots were placed into new eluent for 30 min at room temperature. The eluent was prepared using a 3:1:1 volume ratio of alcohol:acetic acid:glycerin.

### 4.9. qRT–PCR

qRT–PCR was conducted using an ABI 7500 real-time PCR system (Applied Biosystems, Foster City, CA, USA). The Sequence Detection System (SDS) software v.1.4 of the ABI 7500 system was used to analyze the data. The constitutively expressed tubulin gene (*Glyma.19G127700*) was used as a reference gene. Each sample was measured from three technical replicates. The gene expression level was analyzed by the ΔCt program within SDS v.1.4. Primers in Table S1.

### 4.10. Nucleotide Diversity (π) and Level of Genetic Differentiation (F_st_)

The 1295 soybean panel and SNP data used for the genetic diversity and *F*_st_ analysis were described previously [37]. SNPs with missing data >10% or MAF < 5% were filtered, and pairwise genomic differentiation values for wild, landrace and cultivated populations of soybean were calculated using a 10-k-10-k sliding window in VCFtools50.

## Data Availability

All relevant data are included in this paper and its associated Supplementary Information.

## Supplementary Materials

The following supporting information can be downloaded at: https://www.mdpi.com/article/10.3390/ijms23148016/s1.
