# Supplementary material for: Genome-Wide Association Studies Reveal Novel Loci for Herbivore Resistance in Wild Soybean (Glycine soja)
Source: Int J Mol Sci. 2022 Jul 20;23(14):8016. doi: 10.3390/ijms23148016 (PMC9320132; doi:10.3390/ijms23148016)
Supplement: Supplementary file 1 [file ijms-23-08016-s001.zip › ijms-1822306-supplementary.pdf]

Supplementary Information

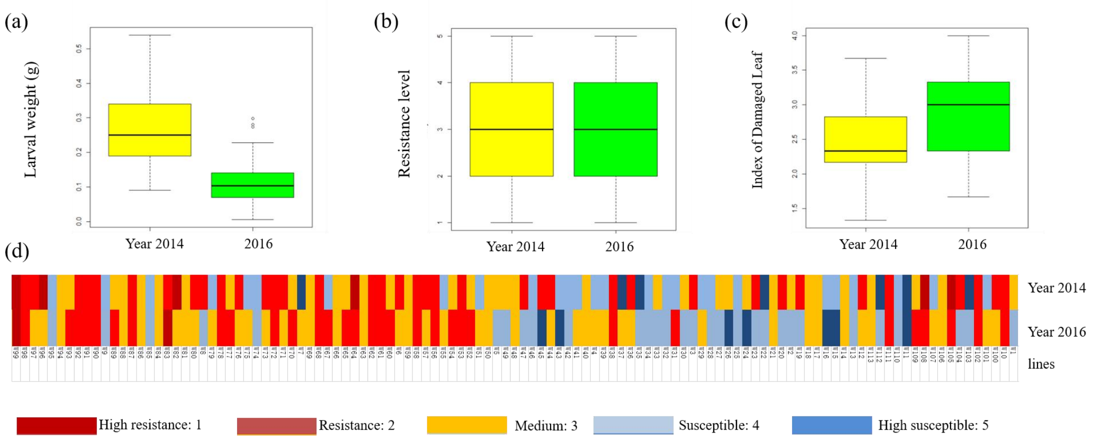

Figure S1: The distribution of larval weight (a), resistance level (b, d) and index of damaged leaf (c) in the two years (year 2014 and 2016).

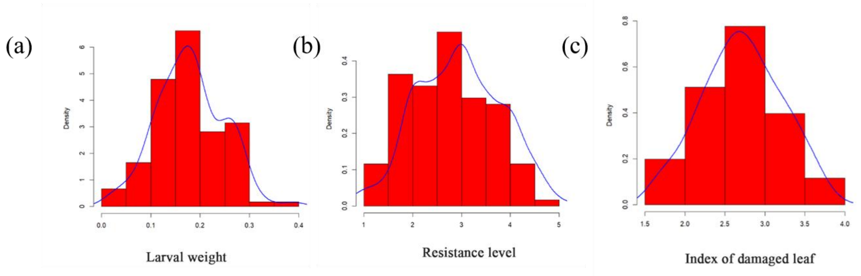

Figure S2: Density distribution of larval weight (a), resistance level (b) and index of damaged leaf (c) with the mean value in two environments.

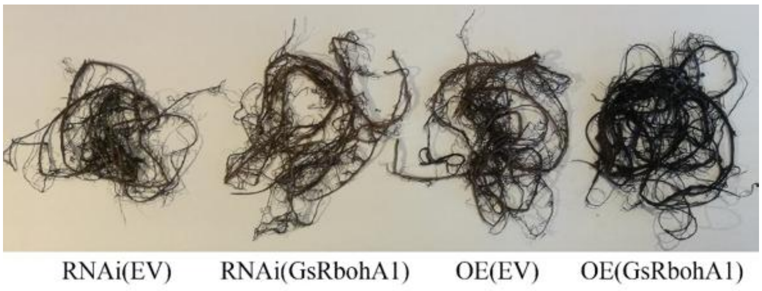

Figure S3: ROS staining of hair roots.

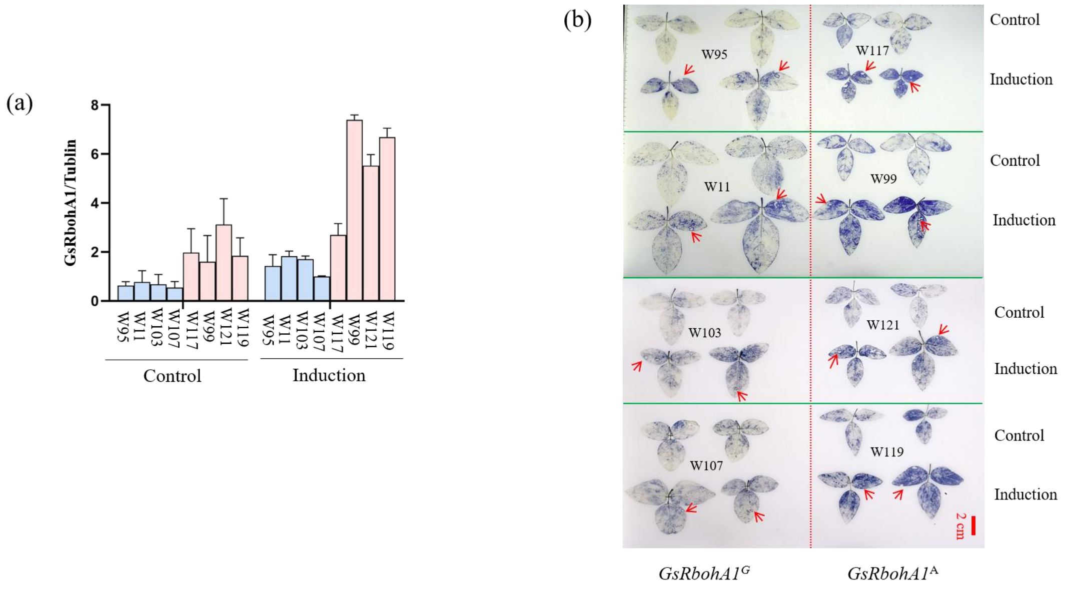

Figure S4: Resistance haplotype *GsRbohA1*^{A} increased *GsRbohA1* expression and ROS communication. (a) Transcriptional level of *GsRbohA1* in *GsRbohA1*^{A} and *GsRbohA1*^{G} haplotypes before and 1 h after common cutworm induction. (b) ROS staining of soybean leaf in *GsRbohA1*^{A} and *GsRbohA1*^{G} haplotypes before and 1 h after common cutworm induction.

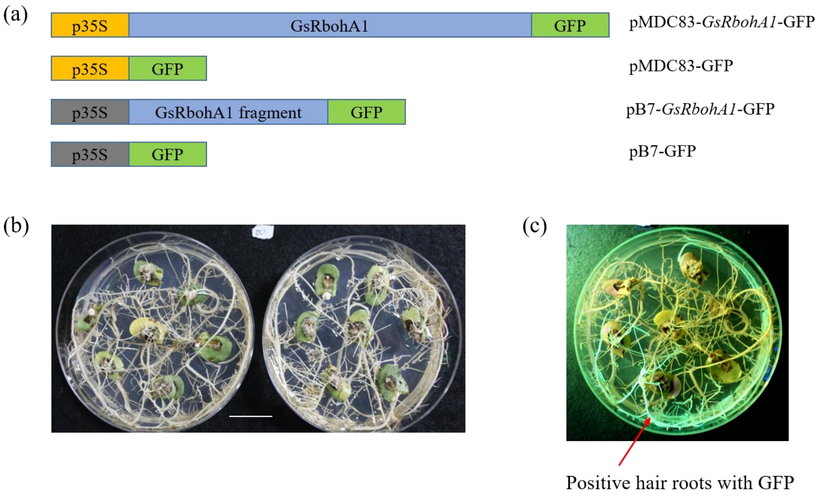

Figure S5: Soybean hairy root transformation. (a) *GsRbohA1*-overexpression and -RNAi plasmids used for soybean hairy root transformation. (b) Soybean hairy roots at 28 days after transformation, scale bar 2 cm. (c) Positive-transformation hairy roots with green fluorescence.

Table S1 List of primer sequences (5'-3') used in this study

Primer name	Primer sequence Forward	Reverse	Vector
Tublin	TTCTCTGCACTCTTCATCAAGCTC	CACACCACTTCCCAGAACTTG	qPCR
GsRbohA1	TGCAAATATCGAAGCCCTCTCA	CAGCTACGGGAGGTTCACAG	
GsRbohA1-p83	CAGGTCGACTCTAGAGGATCCGCCACCATGAACGGTGCTCCCAAG	GGGAAATTCGAGCTCGGTACCTTAGAAATGCTCCTTGTGGA	pMDC83-GsRbohA1-GFP
GsRbohA1-pb7	GGGGACAAGTTTGTACAAAAAAGCAGGCTCCCTGGGAGCGCAGATTCACTA	GGGGACCACTTTGTACAAGAAAGCTGGGTGGCGCCACAATAGAAAACCCC	pB7-GsRbohA1-GFP

Table S2 Phenotypic data of 121 wild soybean accessions in two years

Accession	LW-2014(g)	LW-2016(g)	RL-2014	RL-2016	IDL-2014	IDL-2016
NJAU_W001	0.23	0.179	3	4	2.83	2.33
NJAU_W002	0.27	0.135	3	4	2.67	2.33
NJAU_W003	0.17	0.165	2	4	3.00	3.33
NJAU_W004	0.37	0.086	4	3	2.17	2.00
NJAU_W005	0.23	0.144	3	4	2.50	2.67
NJAU_W006	0.14	0.092	2	3	2.50	3.00
NJAU_W007	0.45	0.110	5	3	2.83	3.67
NJAU_W008	0.19	0.169	2	4	2.33	3.67
NJAU_W009	0.35	0.187	4	4	3.17	3.67
NJAU_W010	0.17	0.074	2	2	2.33	2.33
NJAU_W011	0.54	0.228	5	5	3.17	4.00
NJAU_W012	0.20	0.091	2	3	2.17	2.67
NJAU_W013	0.36	0.140	4	4	2.00	2.67
NJAU_W014	0.24	0.118	3	3	2.50	3.33
NJAU_W015	0.35	0.205	4	5	2.50	4.00
NJAU_W016	0.39	0.273	4	5	3.17	4.00
NJAU_W017	0.27	0.110	3	3	2.33	3.00
NJAU_W018	0.25	0.082	3	3	2.67	3.00
NJAU_W019	0.17	0.145	2	4	1.83	2.33
NJAU_W020	0.16	0.145	2	4	2.33	2.67
NJAU_W021	0.29	0.122	3	3	2.17	3.33
NJAU_W022	0.40	0.144	5	4	2.33	3.33
NJAU_W023	0.17	0.158	2	4	2.17	3.00
NJAU_W024	0.24	0.298	3	5	3.17	3.67
NJAU_W025	0.35	0.148	4	4	2.83	3.67

NJAU_W026	0.29	0.280	3	5	3.33	3.67
NJAU_W027	0.30	0.179	3	4	3.17	3.00
NJAU_W028	0.35	0.169	4	4	2.17	3.67
NJAU_W029	0.37	0.103	4	3	2.33	2.67
NJAU_W030	0.23	0.163	3	4	2.50	3.00
NJAU_W031	0.32	0.066	4	2	2.33	3.00
NJAU_W032	0.37	0.137	4	4	2.33	3.67
NJAU_W033	0.29	0.137	3	4	2.17	3.67
NJAU_W034	0.38	0.139	4	4	2.67	3.67
NJAU_W035	0.47	0.140	5	4	2.00	3.67
NJAU_W036	0.20	0.110	2	3	2.00	4.00
NJAU_W037	0.45	0.114	5	3	2.33	3.00
NJAU_W038	0.18	0.036	2	2	1.83	2.00
NJAU_W039	0.33	0.089	4	3	2.67	3.00
NJAU_W040	0.24	0.121	3	3	2.67	4.00
NJAU_W041	0.33	0.084	4	3	2.17	3.00
NJAU_W042	0.40	0.136	4	4	2.67	3.67
NJAU_W043	0.34	0.216	4	5	2.67	3.33
NJAU_W044	0.17	0.087	2	3	2.50	2.67
NJAU_W045	0.18	0.199	2	5	3.17	4.00
NJAU_W046	0.39	0.174	4	4	2.67	3.67
NJAU_W047	0.21	0.141	2	4	2.17	2.00
NJAU_W048	0.30	0.083	3	3	2.17	3.00
NJAU_W049	0.25	0.154	3	4	3.00	2.33
NJAU_W050	0.27	0.129	3	3	2.00	2.00
NJAU_W051	0.38	0.103	4	3	2.50	2.67
NJAU_W052	0.26	0.038	3	2	2.00	2.33
NJAU_W053	0.18	0.071	2	2	1.67	1.67
NJAU_W054	0.29	0.070	3	2	2.33	3.33
NJAU_W055	0.34	0.085	4	3	3.33	3.33
NJAU_W056	0.22	0.052	2	2	2.67	2.67
NJAU_W057	0.21	0.110	2	3	1.83	2.67
NJAU_W058	0.16	0.065	2	2	2.17	3.33
NJAU_W059	0.28	0.100	3	3	3.67	3.67
NJAU_W060	0.26	0.064	3	2	2.67	2.67
NJAU_W061	0.17	0.042	2	2	2.83	2.33
NJAU_W062	0.21	0.073	2	2	2.33	3.33
NJAU_W063	0.25	0.084	3	3	3.00	4.00
NJAU_W064	0.12	0.067	1	2	3.17	3.33
NJAU_W065	0.28	0.065	3	2	2.50	3.00
NJAU_W066	0.29	0.133	3	3	2.00	3.00
NJAU_W067	0.35	0.041	4	2	2.17	3.00
NJAU_W068	0.18	0.069	2	2	2.00	2.33
NJAU_W069	0.24	0.085	3	3	2.33	3.00

NJAU_W070	0.27	0.077	3	2	2.00	2.67
NJAU_W071	0.20	0.083	2	3	2.17	2.67
NJAU_W072	0.16	0.064	2	2	3.33	2.00
NJAU_W073	0.20	0.082	2	3	2.67	2.00
NJAU_W074	0.36	0.136	4	4	1.83	4.00
NJAU_W075	0.36	0.094	4	3	2.83	3.33
NJAU_W076	0.18	0.110	2	3	3.00	3.00
NJAU_W077	0.27	0.074	3	2	2.83	3.33
NJAU_W078	0.22	0.079	2	2	2.33	3.67
NJAU_W079	0.32	0.106	4	3	1.33	2.33
NJAU_W080	0.21	0.112	2	3	2.00	2.33
NJAU_W081	0.26	0.109	3	3	1.83	2.67
NJAU_W082	0.12	0.106	1	3	2.17	2.67
NJAU_W083	0.16	0.005	2	1	1.50	2.00
NJAU_W084	0.27	0.088	3	3	2.67	2.67
NJAU_W085	0.33	0.146	4	4	2.50	2.00
NJAU_W086	0.24	0.103	3	3	1.33	2.00
NJAU_W087	0.18	0.057	2	2	1.50	1.67
NJAU_W088	0.22	0.134	3	3	1.67	2.33
NJAU_W089	0.29	0.074	3	2	3.17	3.33
NJAU_W090	0.18	0.052	2	2	1.67	2.00
NJAU_W091	0.22	0.052	2	2	2.33	2.67
NJAU_W092	0.18	0.044	2	2	2.33	2.00
NJAU_W093	0.25	0.061	3	2	3.17	3.67
NJAU_W094	0.30	0.088	3	3	3.00	3.33
NJAU_W095	0.37	0.148	4	4	2.83	3.33
NJAU_W096	0.11	0.089	1	3	2.17	2.33
NJAU_W097	0.20	0.081	2	3	2.67	2.33
NJAU_W098	0.15	0.033	2	2	2.83	2.67
NJAU_W099	0.09	0.022	1	1	2.67	2.00
NJAU_W100	0.15	0.119	2	3	2.00	2.67
NJAU_W101	0.39	0.099	4	3	2.67	3.00
NJAU_W102	0.19	0.035	2	2	1.83	2.67
NJAU_W103	0.41	0.137	5	4	2.17	3.67
NJAU_W104	0.20	0.178	2	4	3.17	4.00
NJAU_W105	0.13	0.030	1	2	2.17	2.67
NJAU_W106	0.29	0.096	3	3	2.50	3.33
NJAU_W107	0.40	0.119	4	3	3.00	3.67
NJAU_W108	0.13	0.032	1	2	2.83	3.00
NJAU_W109	0.25	0.036	3	2	2.17	2.67
NJAU_W110	0.37	0.161	4	4	3.67	4.00
NJAU_W111	0.19	0.032	2	2	2.50	2.00
NJAU_W112	0.43	0.175	5	4	1.83	3.67
NJAU_W113	0.24	0.122	3	3	1.50	2.00

NJAU_W114	/	0.131	/	3	/	3.67
NJAU_W115	/	0.104	/	3	/	2.00
NJAU_W116	/	0.151	/	4	/	3.33
NJAU_W117	/	0.046	/	2	/	3.00
NJAU_W118	/	0.036	/	2	/	2.00
NJAU_W119	/	0.023	/	1	/	2.67
NJAU_W120	/	0.022	/	1	/	2.33
NJAU_W121	/	0.056	/	2	/	3.33

Table S3 Descriptive statistics, ANOVA results and broad-sense heritability explained by the population structure for LW, RI and IDL in 121 wild soybean accessions

Trait	Year	Max.	Min.	SD	Mean	CV (%)	ANOVA			h^2(%)
							Y	G	Y*G	
LW (g)	2014	0.5433	0.0900	0.0907	0.2639	34.37	***	***	***	48.86
	2016	0.2983	0.0050	0.0544	0.1074	50.69				
RL	2014	5.00	1.67	0.79	2.98	26.55	***	***	***	49.67
	2016	5.00	2.00	0.76	2.94	25.76				
IDL	2014	3.67	1.33	0.49	2.45	20.18	***	***	***	64.10
	2016	4.00	1.67	0.64	2.95	21.50				

LW larval weight, RL resistance level, IDL index of damaged leaf, Max. maximum, Min. minimum, SD standard deviation, CV coefficient of variation, Y year, G genotype, Y*G interaction of year and genotype, h^2 broad-sense heritability, * P<0.05, * * P<0.01, * * * P<0.001.

Table S4 Significant SNPs associated with LW, RL and IDL

Trait	SNP	Chromosome	Location	P value	-log10P
LW	AX-94083016	11	1448659	6.08E-05	4.216096421
RL	AX-94082508	11	14846	6.61E-05	4.179798541
RL	AX-93785819	11	16557	6.61E-05	4.179798541
RL	AX-93785820	11	18640	6.63E-05	4.178486472
RL	AX-93785821	11	20245	6.34E-05	4.197910742
RL	AX-94082514	11	28297	6.61E-05	4.179798541
RL	AX-93785823	11	28892	6.61E-05	4.179798541
RL	AX-94082517	11	32780	6.61E-05	4.179798541
RL	AX-94286121	11	36107	6.61E-05	4.179798541
RL	AX-93785827	11	38744	6.71E-05	4.17327748
RL	AX-93785828	11	43611	6.61E-05	4.179798541
RL	AX-93785829	11	45025	6.61E-05	4.179798541
RL	AX-93785830	11	46527	6.61E-05	4.179798541
RL	AX-93785831	11	54324	6.50E-05	4.187086643
RL	AX-93785833	11	59512	6.61E-05	4.179798541
RL	AX-94286288	11	63293	6.61E-05	4.179798541
RL	AX-93785835	11	64798	6.61E-05	4.179798541
RL	AX-94082532	11	72758	6.61E-05	4.179798541
RL	AX-94082534	11	76272	6.61E-05	4.179798541
RL	AX-94285858	11	1373761	4.77E-05	4.321481621
RL	AX-93786283	11	1388658	4.77E-05	4.321481621
RL	AX-94083014	11	1430459	4.77E-05	4.321481621
RL	AX-94083021	11	1465715	3.04E-05	4.517126416
RL	AX-93786305	11	1475837	3.04E-05	4.517126416
RL	AX-94083026	11	1478446	3.04E-05	4.517126416
RL	AX-94268530	11	1505923	3.06E-05	4.514278574
RL	AX-93947730	16	7114437	7.61E-05	4.118615343
RL	AX-93848327	16	7120306	7.61E-05	4.118615343
RL	AX-94145475	16	7121795	7.61E-05	4.118615343
RL	AX-94145477	16	7124817	7.61E-05	4.118615343
RL	AX-94145465	16	7106438	7.87E-05	4.104025268
RL	AX-93947728	16	7106675	7.87E-05	4.104025268
RL	AX-93848325	16	7116306	7.87E-05	4.104025268
RL	AX-93816295	13	33244738	8.87E-05	4.05207638
RL	AX-93935497	11	1562584	9.76E-05	4.010550182
IDL	AX-94045571	8	1337507	6.49E-05	4.187755303
IDL	AX-93944925	15	38101535	8.30E-05	4.080921908

The candidate markers mentioned in the text were shaded.
